# Assessing the Perceived Attitude of Nurses towards Childbirth: Experiences of Rural Childbearing Women in Kwara State, Nigeria

**DOI:** 10.4314/ejhs.v35i2.8

**Published:** 2025-03

**Authors:** Adeola Aminat Odebode, Samuel Kolawole Ajiboye, Falilat Anike Okesina, Adijat Mojisola Abdulraheem, Ibrahim Ologele

**Affiliations:** 1 Department of Educational Guidance and Counselling, University of Ilorin, Nigeria University of Ilorin, Nigeria; 2 Department of Health Promotion and Environmental Health, University of Ilorin, Nigeria

**Keywords:** Perceived Attitude, Child Delivery Process, Counsellors, Childbearing Women, Nurses

## Abstract

**Background:**

Childbirth is a crucial event in a woman's life, and healthcare practitioners are expected to approach the process with the appropriate demeanor. This study aimed to investigate the perceived attitude of nurses towards the child delivery process by childbearing women in Kwara State, Nigeria. Additionally, the study examined the influence of moderating variables such as age, educational attainment, and parity on the respondents' views.

**Methods:**

A descriptive survey with a mixed-methods approach was used. A total of 384 childbearing women were selected using simple random and purposive sampling techniques. The participants completed a researcher-designed questionnaire titled the “Perceived Attitude of Nurses Toward Child Delivery Process Questionnaire (PANCDPQ),” with the psychometric properties thoroughly verified. Ten willing participants were also interviewed using an Interview Guide. The data were analyzed using percentages, mean, standard deviation, and Analysis of Variance (ANOVA) at a 0.05 level of significance. Thematic analysis was applied to analyze the qualitative data.

**Results:**

The results indicated that childbearing women in rural areas of Kwara State, Nigeria, perceived nurses as unfriendly towards expectant mothers. They reported that nurses did not provide vital information, failed to give full attention to women in labor, and demonstrated a lack of competence during delivery. The interviews further revealed that nurses spoke recklessly to women in labor and shouted at them.

**Conclusion:**

It was concluded that the perceived attitude of nurses towards the child delivery process by childbearing women in rural areas was inappropriate. It is recommended that nurses be trained on the perceptions of childbearing women regarding their attitudes towards childbirth. Health counsellors should collaborate with nurses to foster the development of appropriate attitudes towards mothers during childbirth, thereby improving service quality.

## Introduction

Nurses play an essential role in healthcare delivery, providing critical support within healthcare institutions. They perform a variety of tasks daily to ensure effective service delivery. Given that there are more nurses than any other healthcare professionals, it is vital for them to adopt a professional framework to guarantee quality care (1). Adopting core values helps ensure that nurses fulfill their duties effectively and efficiently while ensuring patient safety. Nurses are also expected to adhere to the Department of Health's 6 Cs of nursing (2), which include: (i) courage, (ii) communication, (iii) competence, (iv) compassion, (v) commitment, and (vi) care. These principles should be particularly followed when assisting women during childbirth.

Child delivery is a significant event in a woman's life, and every woman deserves appropriate care during labor and delivery. The World Health Organization (WHO) has emphasized that the quality of interaction between women and healthcare providers is crucial for positive childbirth outcomes (3). In this context, Respectful Maternity Care (RMC) has emerged as a key strategy for improving the quality and utilization of maternity care (4). RMC emphasizes women's fundamental rights, the unique needs of women and newborns, and equitable access to evidence-based care during and after childbirth (4).

The childbirth process involves the expulsion of the fetus and placenta from the womb and is characterized by a series of continuous uterine contractions that help in the thinning and expansion of the cervix, allowing the fetus to pass through the birth canal (6). Childbirth can occur through vaginal delivery or cesarean section (7). The delivery process is categorized into three stages: the first stage involves cervical changes and ends when the cervix is fully dilated; the second stage, known as the “pushing stage,” ends with the birth of the baby; and the third stage concludes with the delivery of the placenta (8, 9, 12).

According to Kirby, Kirby, and Frost (16), vaginal delivery is the most common and safest method of childbirth. In certain circumstances, tools such as forceps or a vacuum may be used to assist in the delivery. Cesarean sections may also be necessary when vaginal delivery is not possible. Childbirth is often accompanied by intense pain for the expectant mother.

Women require encouragement and support during labor. Despite the ongoing efforts by nurses to enhance care quality, many women continue to report dissatisfaction with nurses' attitudes. The relationship between the density of healthcare workers and maternal mortality rates in Nigeria remains imbalanced (17, 18). Over one-third of births in Nigeria are attended by skilled health professionals, but negative attitudes and behaviors from healthcare providers can hinder maternal and infant health promotion efforts, undermining women's right to respectful care (17, 19, 20).

In 2015, an estimated 303,000 maternal deaths occurred globally, with 99% of them in low- and middle-income countries. Nigeria accounted for 19% of global maternal deaths, with 58,000 deaths annually between 2015 and 2018 (21, 22). A key factor contributing to the high maternal mortality rate is the low rate of skilled birth attendance, with only 45% of births attended by trained personnel (21, 23). Skilled care during pregnancy and childbirth has been shown to significantly reduce maternal and infant mortality (22).

Liu et al. (24) reported that women delivering in health facilities often face mistreatment, despite the advocacy for institutional delivery to improve maternal and newborn health outcomes. In Nigeria, a suboptimal rate of institutional delivery continues, with many women citing poor-quality services as the reason for not delivering in health facilities. This poor care experience shapes mothers' perceptions of healthcare providers and affects their health-seeking behavior.

In rural areas of Kwara State, where fewer people reside and resources are limited, there is a notable lack of formal education, with many people engaged in farming and petty trading. These women often lack confidence and awareness. Particularly in rural Nigeria, many pregnant women experience mistreatment, abuse, and neglect during labor. The behavior of healthcare providers can either build or erode trust among pregnant women. Previous studies have shown that nurses' attitudes contribute to complications and even maternal and infant deaths (25, 26). Despite previous research on nurses' attitudes towards childbirth, there is limited focus on rural areas in Kwara State, which justifies this study.

This study aimed to address the research question: What is the perceived attitude of nurses towards the child delivery process by childbearing women in rural areas of Kwara State?

The study also tested the following hypotheses:

1. There is no significant difference in the perceived attitude of nurses towards the child delivery process by childbearing women in rural areas of Kwara State based on age.
2. There is no significant difference in the perceived attitude of nurses towards the child delivery process by childbearing women in rural areas of Kwara State based on educational attainment.
3. There is no significant difference in the perceived attitude of nurses towards the child delivery process by childbearing women in rural areas of Kwara State based on parity.

## Methods

The purpose of this study was to assess the perceived attitude of nurses towards the child delivery process by childbearing women in rural areas of Kwara State, Nigeria. A convergent mixed-methods approach was used, involving both quantitative and qualitative data collection, analyzed separately before merging the results for comprehensive analysis. This approach was deemed necessary to accurately capture the nurses' attitudes as perceived by childbearing women and to understand their experiences. The study was conducted from March to July 2023.

**Population and sample**: Due to the lack of a specific population registry, Cochran's formula was used to determine the sample size, resulting in 384 participants after accounting for a 30% increase to offset potential losses. Participants were selected based on the following criteria: women aged 18 and older, who had delivered within the last six months, were healthy and resided in rural areas of Kwara State. Women who were sick, or who declined participation were excluded.

A multi-stage sampling technique was employed as follows:

All 16 local government areas (LGAs) in Kwara State were included to ensure equal representation. Two rural areas were selected randomly from each LGA, resulting in a total of 32 rural areas. One maternity center was selected purposively from each rural area due to the high number of childbirths. An average of 12 childbearing women were selected from each maternity center to reach the sample size of 384.

**Instrument**: A researcher-designed questionnaire titled “Perceived Attitude of Nurses towards Child Delivery Process Questionnaire (PANCDPQ)” was used to collect quantitative data from childbearing women in rural Kwara State. The questionnaire consisted of two sections: Section A gathered demographic information (age, educational attainment, and parity), while Section B contained 15 items assessing respondents' perceptions of nurses' attitudes toward the childbirth process. An Interview Guide, consisting of 5 questions, was used for qualitative data collection.

The PANCDPQ was validated by experts in counseling and nursing, and its reliability was confirmed with a Cronbach's Alpha of 0.89. The questionnaire used a 4-point Likert scale, where percentage was used for the responses. Scores between 50% and above indicated a friendly attitude and scores below 50% indicated an unfriendly attitude.

**Data collection and analysis**: The questionnaire forms were administered to the selected respondents, who were informed of the study's purpose and assured of the confidentiality of their responses. Completed questionnaires were collected immediately to ensure full retrieval. Interviews were conducted at each maternity center, each lasting approximately 60 minutes. The demographic data were analyzed using percentages, while ANOVA was used to test the null hypotheses. Thematic analysis was applied to the interview data, with the researchers listening to the recordings multiple times to extract meaningful insights.

## Results

**Demographic data**: A total of 384 questionnaire forms were administered; however, only 350 were completely filled, yielding a 91% return rate, which fell within the calculated sample. The frequency distribution of the demographic data showed that 96 (27.4%) respondents were between the ages of 18–28 years, 130 (37.1%) were between 29–39 years, 72 (20.6%) were between the ages of 40–49 years, and 52 (14.9%) were aged 50 years and above. Similarly, 155 (44.3%) of the respondents had no formal education, 150 (42.8%) had either a Primary or Secondary School Certificate, 42 (12.0%) had a first degree, and 3 (0.9%) had a postgraduate degree. Furthermore, 17 (4.9%) respondents had 1 child, 73 (20.9%) had 2 children, 154 (44.0%) had 3 children, and 106 (30.2%) had 4 or more children.

**Research question**: *What is the attitude of nurses towards the child delivery process as perceived by childbearing women in rural areas?*

Table 1 presents a ranked summary of respondents' perceptions of nurses' attitudes during labor, based on specific aspects of care. The items are listed in descending order of importance, determined by the scores assigned to each aspect. The scores reflect the extent to which respondents agreed with the statements, with higher scores indicating more positive experiences. Encouragement to cope with labor pain ranked the highest (48.0), indicating that this was the most positively perceived aspect of the nurses' behavior. Friendliness toward me as an expectant mother (46.0) and giving me total attention as I needed (44.0) were also highly rated, suggesting that interpersonal aspects of care were valued. Displaying competence in attending to me during labor scored the lowest (27.0), indicating a perceived gap in technical or professional skills during the labor process. Items related to communication, such as taking time to communicate with me at every stage of the process (30.0), were ranked lower, highlighting potential areas for improvement in information sharing.

**Table 1:** Standardized Response Index (SRI) and Rank Order of the Respondents

Item No.	Nurses displayed the following to me when I was in labor	SRI (%)	Rank Order
11	Encouragement to cope with labor pain	48.0	1^st^
1	Friendliness to me as an expectant mother.	46.0	2^nd^
7	Giving me total attention as I needed	44.0	3^rd^
10	Giving me adequate information about child delivery process	43.0	4^th^
6	Involving me in every intervention during labor	42.0	4^th^
2	Appropriate care in order to lessen my worries	40.0	6^th^
15	Presenting care in manners that encourages referrals	39.0	7^th^
9	Carrying out their duties during child delivery in the most diligent ways	38.0	8^th^
3	Being humorous when I feel despair	36.0	9^th^
5	Showing compassion when I was in labor pain	35.0	10^th^
13	Giving adequate information about medication	33.0	11^th^
4	Taking time to communicate to me at every stage of the process	30.0	12^th^
12	Ensuring that I enjoy full comfort	28.6	13^th^
14	Treating me in ways that repose huge confidence in them	27.2	14^th^
8	Displaying competence in attending to me during labor	27.0	15^th^

Table 2 shows the distribution of respondents' attitudes in the study. Out of the total 350 respondents, 122 (34.86%) perceived that nurses exhibited a friendly attitude towards them during the child delivery process, while 228 (65.14%) perceived that nurses exhibited an unfriendly attitude. This indicates that the majority of the respondents perceived that nurses exhibited an unfriendly attitude towards women during the child delivery process, with only about two-fifths expressing that nurses displayed a friendly attitude.

**Table 2:** Respondents' Expression of the Attitude of Nurses towards Child Delivery Process

Category	No of Respondents (%)
Friendly Attitude	122 (34.9)
Unfriendly Attitude	228 (65.1)
Total	350 (100)

**Hypotheses testing**: This section presents the results of the inferential analysis carried out on the data. Three null hypotheses were formulated and tested for this study. These hypotheses were tested using Analysis of Variance (ANOVA) and the student t-test at an alpha (0.05) level of significance.

**Hypothesis 1**: *There is no significant difference in the attitude of nurses towards the child delivery process as perceived by childbearing women in rural areas based on age.*

Table 3 shows the result of the analysis based on the age of the respondents. It can be seen from the table that the calculated F value (0.14) is less than the critical F value (2.60). Since the p-value (0.85) is greater than the level of significance (0.05), the hypothesis is not rejected.

**Table 3:** Analysis of Variance (ANOVA) Showing Respondents' Expression of the Attitude of Nurses towards Child Delivery Process Based on Age

Source	df	SS	MS	Cal.F-ratio	F-Crit. Ratio	P
Between Groups	3	0.469	0.156	0.14	2.60	0.85
Within Groups	346	385.904	1.110			
Total	349	386.373				

**Hypothesis 2**: *There is no significant difference in the attitude of nurses towards the child delivery process as perceived by childbearing women in rural areas based on educational attainment.*

Table 4 shows the result of the analysis based on the educational attainment of the respondents. The calculated F value (0.74) is less than the critical F value (2.60). Since the p-value (0.58) is greater than the level of significance (0.05), the hypothesis is not rejected.

**Table 4:** Analysis of Variance (ANOVA) Showing Respondents Expression of the Attitude of Nurses towards Child Delivery Process Based on Education

Source	df	SS	MS	Cal.F-ratio	F-Crit. ratio	F- Sig.
Between Groups	3	2.478	0.826	0.74	2.60	0.58
Within Groups	346	383.895	1.111			
Total	349	386.373				

**Hypothesis 3**: *There is no significant difference in the attitude of nurses towards the child delivery process as perceived by childbearing women in rural areas based on parity.*

Table 5 shows the result of the analysis based on the number of children (parity) of the respondents. The calculated F value (0.51) is less than the critical F value (0.74). Since the p-value (0.85) is greater than the level of significance (0.05), the hypothesis is not rejected.

**Table 5:** Analysis of Variance (ANOVA) Showing Respondents Expression of the Attitude of Nurses towards Child Delivery Process Based on Parity

Source	df	SS	MS	Cal.F-ratio	F-Crit. ratio	F- Sig.
Between Groups	3	1.720	0.573	0.51	2.60	0.74
Within Groups	346	384.653	1.112			
Total	349	386.373				

**Results from the interview**: Several questions in line with the objectives of the study were asked during the interview. However, only relevant information is provided in this section. The recordings were reviewed multiple times to fully understand the meaning behind the participants' responses. The responses are presented without modification, and six out of the ten interviews are included. The main points from the interview responses are as follows:

Question: *How would you describe nurses' interactions with you during labor?*

Participant No. 1: First Degree, Number of Children: 3, Age: 36 years.

Response: *“Well, the nurses were not too friendly with me at all when I was about to give birth to my baby. I felt they needed to take it easy with women in labor.”*

Participant No. 4: First Degree, Number of Children: 5, Age: 40 years.

Response: *“As a matter of fact, the nurse who attended to me was slapping and beating me all in the name of helping me. I am not happy with the way the nurse handled me. If I must say, they were very unkind to me.”*

Participant No. 8: No Formal Education, Number of Children: 2, Age: 28 years.

Response: *“Nurses have a negative attitude, of course. The nurses are not like the doctors. The nurses have negative ways of relating to us when we want to give birth. The policymakers should help us tell them and caution them.”*

Participant No. 9: First Degree, Number of Children: 2, Age: 32 years.

Response: *“I don't like the way nurses talk to women in the hospital. They shout at us during labor as if they have never gone through the pain of childbirth. They act as if they do not have human feelings. I would say they have a negative attitude.”*

Participant No. 10: Primary/Secondary School Holder, Number of Children: 4, Age: 42 years.

Response: *“I have been looking for a place to pour my mind. The day I gave birth was not funny. The nurses just left me in the labor room without telling me where they were going or why they left. I was shouting and screaming for help when the baby was coming. Luckily, a cleaner came in and helped me to call them. Nurses are not nice towards women during childbirth.”*

Participant No. 7: First Degree, Number of Children: 1, Age: 24 years.

Response: *“I told the nurse to give me an injection so I wouldn't feel the pain of the suture, but she refused! I cried my heart out; I felt the pain of the suture more than the delivery pain. I can never forget this experience. The attitude of nurses is nothing to write home about.”*

Question: *Can you share specific examples of either positive or negative interactions with the nurses?*

Response 1: *“During labor, I asked for help because I was in severe pain, but the nurse dismissed me, saying I should stop complaining because every woman goes through it. I felt so helpless.”*

Response 2: *“The nurse was shouting at me to push faster, and when I told her I was tired, she said I was being lazy. It made the entire experience more stressful.”*

Response 3: *“I was struggling to move because of the contractions, and instead of helping me, the nurse scolded me for not following her instructions quickly enough. It felt humiliating.”*

Response 4: *“When I asked a question about the procedure they were about to do, the nurse just ignored me and continued without explaining. It made me feel like my concerns didn't matter.”*

Question: *How would you rate the overall attitude of the nurses during your labor on a scale of 1 to 10?*

Response 1: *“I would give them a 3. They were more focused on completing their tasks than providing any form of comfort or support.”*

Response 2: *“Honestly, I'd rate their attitude a 2. They were impatient and treated me as if I was a burden instead of someone in need of care.”*

Response 3: *“I'd say a 4. While they did their job, they showed no empathy or effort to make the experience less frightening for me.”*

Response 4: *“I would give them a 1. The way they spoke to me was harsh, and they showed no compassion throughout the labor process.”*

## Discussion

The findings of this study revealed that nurses are perceived to have unfriendly attitudes towards women in labor. Respondents reported that nurses were not friendly, did not offer full attention to women in labor, failed to encourage women to cope with labor pain, did not take the time to communicate with women during each stage of the labor process, and did not ensure that women enjoyed full comfort. Furthermore, nurses were seen as lacking competence in their care during labor. These perceptions were reinforced by the interviews, where all participants expressed that nurses displayed unfriendly attitudes. For example, one participant commented: *“The nurses are not like the Doctors, the nurses have negative ways of relating with us when we want to put to bed. The policy makers should help us tell them and caution them. Nurses have negative attitude of course.”* Another participant stated: *“I don't like the way nurses talk to women in the hospital. They shout at women during labor as if they have never gone through the pain of childbirth. They act as if they do not have human feelings. I will say they have negative attitude.”* This clearly indicates that, as perceived by the participants, nurses exhibit unfriendly attitudes during the child delivery process.

This finding contradicts the conclusions of (3), which suggested that nurses maintain a positive attitude towards women during labor. In their study, nurses were described as readily available to women, showing kindness and compassion towards their concerns and pain. Similarly, nurses were found to be kind, polite, and sympathetic in their approach to women in labor. However, this study's findings align with the work of (32, 33, 34, 35), which reported that nurses engage in verbal abuse, shouting, scolding, and using insulting language during labor. The results of this study suggest that nurses may lack patience, empathy, and encouragement, leading respondents to perceive their attitudes as unfriendly.

The findings also revealed no significant difference in the attitudes of nurses towards the child delivery process as perceived by childbearing women in rural areas of Kwara State, based on age. This suggests that the perception of nurses' attitudes does not vary across different age groups. Women of varying ages seemed to experience similar treatment during labor, regardless of their age. This result supports McNiven *et al.* (35), which found that nurses treated women in labor similarly, regardless of their age. The study posits that all women, irrespective of age, undergo a similar degree of pain during childbirth, which could explain why their perceptions of nurses' attitudes do not differ significantly based on age.

Furthermore, the study found no significant difference in the attitudes of nurses towards the child delivery process based on education. This implies that women in labor, irrespective of their educational background, were treated the same way by nurses. This finding contradicts the work of Okafor *et al.* (4), which indicated that education level influenced the way mothers were treated during the child delivery process. It was expected that educational attainment would affect respondents' perceptions of nurses' attitudes. However, in this study, women's education did not appear to influence how they were treated during labor. This could suggest that nurses lack the skills or experience to treat women differently based on education, or that they exhibit a negative attitude towards all patients, regardless of their educational background.

The study also showed no significant difference in the attitude of nurses towards child delivery based on parity. This means that women in labor, whether it was their first childbirth or not, were treated the same way by nurses. This contradicts findings from other studies, such as one that reported discrimination based on high parity, where mothers were mocked by nurses due to the number of children they had (19). The results suggest that nurses may lack compassion and do not provide preferential treatment based on parity, resulting in similar responses from respondents regardless of their number of children.

In conclusion, it is evident that nurses display inappropriate attitudes towards the child delivery process, as perceived by childbearing women in rural areas. Nurses are reported to be unfriendly, failing to give full attention to women in labor, neglecting to communicate health information appropriately, and showing insufficient empathy. Furthermore, no significant differences were found in the attitudes of nurses based on the age, educational background, or parity of the women.

Based on the findings, the following recommendations are made:

- Hospital administrators should introduce a feedback form for women to fill out after childbirth. This form should evaluate the nurses' attitudes during the delivery process, ensuring that both friendly and unfriendly are addressed. This feedback will help improve nurses' attitudes, particularly in rural areas.
- Nurses should provide education to expectant mothers during antenatal care, informing them about the various gestures they might encounter during childbirth. This will help prevent misunderstandings about the nurses' actions, ensuring that women do not misinterpret care as unkindness. For example, slapping a mother's thigh during delivery to prevent her from closing her legs should not be perceived as a negative act.
- Refresher courses should be designed for nurses in rural areas to continually improve their attitudes and interpersonal skills, ensuring they maintain a friendly and empathetic approach towards women in labor.
- Health counsellors should organize awareness workshops in rural areas to educate both nurses and mothers about the attitudes and perceptions surrounding labor care. This awareness can foster positive changes in behavior. Nurses should be made aware that their attitudes significantly impact the safety of both mothers and babies during childbirth. Health counselors should work on improving the attitudes of nurses in rural areas to enhance the overall quality of healthcare services provided.
